# Genome-wide associations of signaling pathways in glioblastoma multiforme

**DOI:** 10.1186/1755-8794-6-11

**Published:** 2013-03-28

**Authors:** Stefan Wuchty, Alexei Vazquez, Serdar Bozdag

**Affiliations:** 1 National Center of Biotechnology Information, National Library of Medicine, National Institutes of Health, Bethesda, MD, 20894, USA; 2 Department of Radiation Oncology, The Cancer Institute of New Jersey and University of Medicine and Dentistry of New Jersey, Robert Wood Johnson Medical School, New Brunswick, NJ, 08901, USA; 3 Dept. of Mathematics, Statistics and Comp. Science, Marquette University, Milwaukee, WI, 52333, USA; 4 National Cancer Institute, National Institutes of Neurological Disorder and Stroke, National Institutes of Health, Bethesda, MD, 20892, USA; 5 Present address: Dept. of Neuroscience, Mayo Clinic, Jacksonville, FL, 32224, USA

## Abstract

**Background:**

eQTL analysis is a powerful method that allows the identification of causal genomic alterations, providing an explanation of expression changes of single genes. However, genes mediate their biological roles in groups rather than in isolation, prompting us to extend the concept of eQTLs to whole gene pathways.

**Methods:**

We combined matched genomic alteration and gene expression data of glioblastoma patients and determined associations between the expression of signaling pathways and genomic copy number alterations with a non-linear machine learning approach.

**Results:**

Expectedly, over-expressed pathways were largely associated to tag-loci on chromosomes with signature alterations. Surprisingly, tag-loci that were associated to under-expressed pathways were largely placed on other chromosomes, an observation that held for composite effects between chromosomes as well. Indicating their biological relevance, identified genomic regions were highly enriched with genes having a reported driving role in gliomas. Furthermore, we found pathways that were significantly enriched with such driver genes.

**Conclusions:**

Driver genes and their associated pathways may represent a functional core that drive the tumor emergence and govern the signaling apparatus in GBMs. In addition, such associations may be indicative of drug combinations for the treatment of brain tumors that follow similar patterns of common and diverging alterations.

## Background

Gliomas represent a heterogeneous family of primary brain tumors that are a significant cause of cancer mortality in the United States [1] with glioblastoma multiforme (GBM) as their most aggressive form. While gliomas strongly differ in their geno- and phenotype, genetic and molecular heterogeneities contribute to the biological and clinical behaviour of different glioma subtypes. The availability of high-throughput gene expression profiles [2-4] provided the opportunity for a quantitative characterization of individual tumors and their classification [5-7]. Recently, several groups have identified subnetworks and pathway-based features that are associated with certain GBM types [8-11] as well as utilized interactions to identify driver genes [12].

The genomic set-up of GBMs is increasingly well characterized [11,13,14], allowing the identification of certain signature alterations. In addition, correlations between changed expression levels of genes and their corresponding genomic alterations are currently investigated [15,16]. However, genomic profiling poses a significant challenge to uncover driving genomic alterations from the large number of deletions and amplifications present in cancer genomes.

The use of microarray technology to simultaneously measure expression of many different genes has been a driving force for the systematic mapping of eQTLs [17,18], since gene expression in many individuals is the substrate for investigating the effects of genomic changes on the expression of individual genes. While some eQTL analyses of human brain tissue have been recently reported [19], eQTL studies have also been combined with network analyses to identify transcription modules of disease-related, co-expressed genes [20-23] and to find causal pathways in glioblastomas [24].

To account for the observation that biological functions are mediated by groups of genes, we determined associations between the expression of pathways and genomic copy number alterations with a machine learning approach. While large signature alterations were driving the association patterns of over-expressed pathways, we found the opposite for under-expressed pathways, an observation that held for composite effects between chromosomal alterations as well. Confirming their biological relevance, identified regions were enriched with driver genes that play a role in gliomas. As a consequence, we observed pathways that were significantly enriched with such driver genes. We conclude that such pathways may indicate a functional core that governs the signaling machinery and tumor emergence in GBMs.

## Results

### Determination of pathway associations

**Figure 1:**
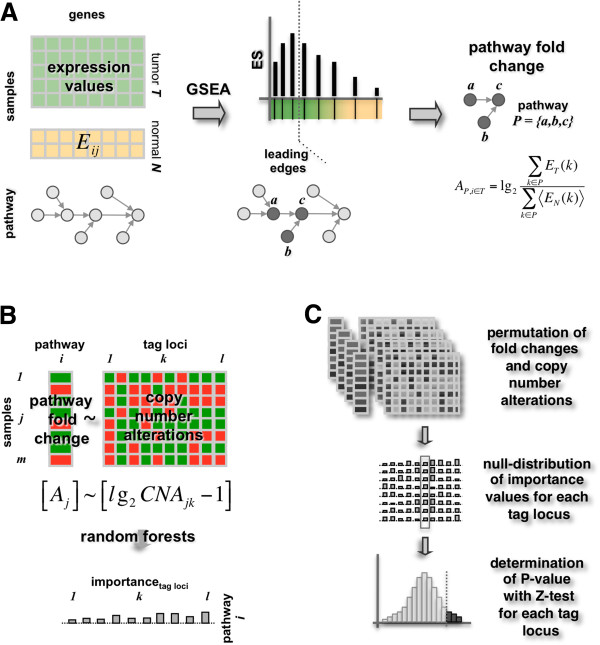
**Outline of the procedure.** (**A**) We used 158 gene expression samples of Glioblastoma Multiforme patients (GBM) and 21 non-tumor control samples. Using 181 pathways we applied Gene Set Enrichment Analysis (GSEA) and found 119 over- and 62 under-expressed pathways. Determining leading edge genes that govern their over/under expression when comparing disease to control cases we represented each pathway by its expression fold change score. (**B**) We fitted pathway fold change scores as a function of the corresponding copy number variations of tag-loci. Using random forest algorithm we obtained a normalized importance score of each locus/pathway pair. (**C**) To assess the statistical significance of a tag-locus’ importance for fitting a pathways expression we performed permutation tests by randomizing both pathway fold change scores and copy number alterations. Focusing on such random distributions of importance scores we applied a Z-test to determine P-values, allowing us to assess the significance of an association between each tag-locus and pathway.

We used gene expression profiles of 158 Glioblastoma Multiforme (GBM) patient and 21 non-tumor control samples from epilepsy patients that were collected from the NCI-sponsored Glioma Molecular Diagnostic Initiative (GMDI) and from Henry-Ford hospital (HF) [13,25]. Accounting for the observation that genes perform their biological functions as an assembly of genes rather than in isolation, we collected 181 signaling pathways from the PID database [26]. Utilizing Gene Set Enrichment Analysis (GSEA) [27] we compared GBM to non-tumor control samples and found 119 over-expressed pathways with a positive enrichment score. Moreover, we obtained 62 under-expressed pathways with a negative enrichment score. We further determined subsets of genes in each signaling pathway that govern the pathways over/under expression in the disease cases (Figure 1A). Such ‘leading edge genes’ were defined as subsets of genes that appappeared in an expression ranked gene list before the enrichment score of a given pathway reached its maximum [27]. Representing each pathway by its corresponding set of leading edge genes we assigned a sample specific expression fold change score to each pathway. In particular, we defined such a score of pathway *p* in disease sample *j* as $A_{p,j}=lg_{2}\frac{\sum {}_{i\in p}E_{i,j}}{\sum {}_{i\in p}\langle E_{i}^{N}\rangle }$, where *E*_*i,j*_ is the expression value of gene *i* in disease sample *j,* and $\langle E({}_{i}^{N}\rangle )$ is the average expression of gene *i* in the set of control samples (Figure 1A).

Searching for genomic loci that potentially play a role in the underlying expression phenotype, we determined associations between the expression fold change scores of pathways and copy number variations of genomic loci. Since genomic variations in neighboring regions tend to be highly correlated, we first chose a subset of 1,510 representative loci (*i.e.* tag-loci) in GBMs. Specifically, we represented each locus as a *x*-dimensional vector of copy number alterations in the corresponding *x* = 158 patient samples. Focusing on a potential tag-locus, we greedily accumulated all consecutive loci, so that the Pearson’s correlation coefficient of any consecutive loci in the region was > 0.95 [24]. While the number of genes a tag-locus can harbor varied strongly we found an average of 6.1 genes per tag-locus, a number that is comparable to the median of 6.5 genes in pooled analyses of human cancers [14].

We searched for genome-wide associations by non-linearly fitting pathway fold change scores as a function of tag-loci’s specific copy number alterations in all GBM samples (Figure 1B). We represented copy number alterations *CNA* of a tag-locus *i* in sample *j* as *lg*_2_*CNA*_*i,j*_ and applied random forest algorithm to assess the impact of a tag-locus on the regression process by its normalized importance score. Reflecting the increase of the prediction error when the given locus is omitted in the regression process, we defined the normalized importance as ${\bar{I}}_{l}\left(p\right)=\frac{I_{i}\left(p\right)}{\sigma_{i}\left(p\right)}$, where *I*_*i*_*(p)* is the average importance, and *σ*_*i*_*(p)* is the standard error of a tag-locus *i* for a given pathway *p*.

To assess the statistical significance of the normalized importance of each locus and pathway pair we randomized sample-specific pathway fold changes and copy number alterations. We applied a Z-test to null distributions thus obtained (Figure 1C) and calculated a P-value for each tag-locus/pathway pair. Correcting all P-values by their corresponding false-discovery rate [28] we used FDR < 0.05 as a threshold to define a significant association. While we found 504 significant associations between 109 over-expressed pathways and 267 tag-loci we observed 471 associations between 56 under-expressed pathways and 209 tag-loci (Additional file 1 Table S1).

### Analysis of associations

**Figure 2:**
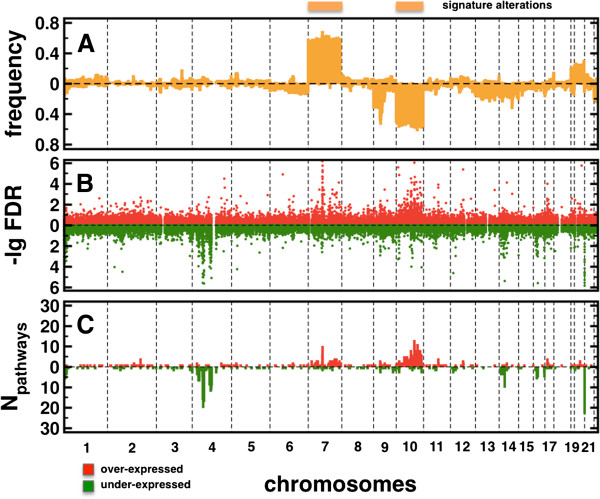
**Statistics of associations.** (**A**) The profile of genomic alterations in glioblastomas allowed us to observe large areas of genomic amplifications on chromosome 7 and deletions on chromosome 10. (**B**) Considering their significance, we found that associations to over-expressed pathways largely coincided with signature alterations. In turn, strong associations to under-expressed pathways mostly appeared on chromosome 4. (**C**) Such observations were emphasized by the number of different pathways that tag-loci were associated with if the FDR of an association was < 0.05.

As a benchmark we show a profile of genomic alterations in glioblastomas in Figure 2A. Specifically, we determined the frequency of patients with |*CNA*_*i*_| > 1.5 at each tag-locus *i*, allowing us to observe large signature areas of genomic amplifications on chromosome 7 and deletions on chromosome 10. In Figure 2B, we show the distribution of FDRs of all tag-locus/pathway associations. While associations to over-expressed pathways largely coincided with signature alterations, we observed strong associations to under-expressed pathways that mostly appeared on chromosome 4. PDGFRA*,* KIT*,* and KDR genes that are located on the amplified segment 4 q12 probably play an important role in tumor biology due to their increased expression of receptors and their ligands. Specifically, Imatinib mesylate targets PDGF receptors while KIT was indicated as a mediator of anti-tumor activity in patients with recurrent GBM [29]. Such results were confirmed in Figure 2C where we plotted the number of different pathways that were significantly associated with tag-loci (FDR < 0.05).

**Figure 3:**
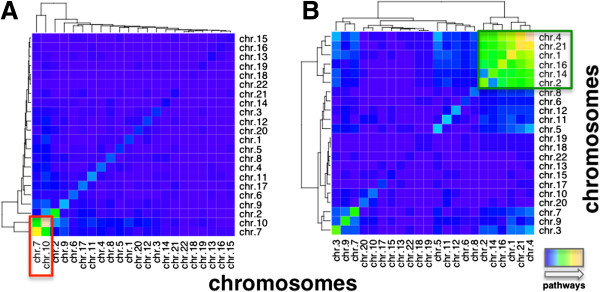
**Chromosomal analysis of significant associations.** In (**A**) we counted the number of different, over-expressed pathways that were significantly associated with tag loci on given chromosomes in GBMs. Clusters in the heatmap suggested that chromosomes 7 and 10 largely shared most pathways (red box). (**B**) Analogously, we determined such overlaps of under-expressed pathways, indicating a more scattered result. Chromosomes 1, 2, 4, 14, 16 and 21 appeared to strongly share pathways (green box).

Determining associated genomic areas we counted the number of pathways that mutually shared tag-loci. We binned loci according to their corresponding chromosomes and pooled all pathways that were significantly associated with tag-loci on the corresponding chromosomes. We observed a pronounced cluster of chromosomes, pointing to genomic alterations that were associated to the same overlapping sets of over-expressed pathways (Figure 3A). Specifically, we found that most pathways were shared between tag-loci on chromosomes 7 and 10. In turn, tag-loci on chromosomes 1, 2, 4, 14, 16 and 21 shared numerous under-expressed pathways, suggesting that composite effects between associated tag-loci largely follow the initial patterns of single associated loci (Figure 3B).

### GRAIL analysis

Since each tag-locus on average harbored more than 6 genes we used GRAIL algorithm [30] to investigate the relevance of such identified genomic regions based on previous knowledge about glioma specific disease regions. Utilizing co-reports of genes in PubMed abstracts, GRAIL explores genes in candidate and reference genomic regions and automatically assesses their degree of relatedness. As references we used a list of genes that are commonly altered in gliomas [2,31] (Additional file 2 Table S2), allowing us to identify potential candidate (driving) genes. As for associated over-expressed pathways, we found 87 tag-loci with genes that were significantly similar to genes in the reference regions and associated to over-expressed pathways (GRAIL P-value < 0.05, Additional file 3 Table S3). In turn, we found 67 such tag-loci with associated under-expressed pathways (Additional file 3 Table S3). In particular, we show such loci that were associated to more than one pathway in Table 1. Generally, tag-loci that were associated to many pathways were highly enriched with genes that were previously reported to have a driving role in the biology of brain tumors. Qualitatively, genes that were associated to over-expressed pathways included prominent signaling and regulation genes that are involved in receptor tyrosine kinase (RTK) signaling (EGFR, EGF, KRAS, PTEN, FRAP1, PIK3 subunits and NF1). In particular, the RTK pathway plays a role in the mediation of growth signals to enhance cell survival and proliferation. The most commonly affected gene in the RTK pathway is EGFR, which is amplified in as many as 45% of GBMs resulting in increased mRNA expression [2,32]. Other RTKs were also shown affected in GBMs, such as amplification of PDGFRA and cMET in 13% and 4%, respectively, and mutation of ERBB2 in 8% of cases [2].

**Table 1:** GRAIL analysis of associations to over- and under-expressed pathways of GBMs

**Over-expressed pathways**				**Under-expressed pathways**			
**Tag-locus**	**Chr.**	**N**_**pw**_	**Gene**	**Tag-locus**	**Chr.**	**N**_**pw**_	**Gene**
SNP_A-1731917	10	13	PTEN	SNP_A-1705677	4	17	TMPRSS11A
SNP_A-1656043	7	10	EGFR	SNP_A-1668058	4	11	BMPR1B
SNP_A-1742783	10	8	PLCE1	SNP_A-1654343	4	9	EGF
SNP_A-1662548	10	8	HABP2	SNP_A-1669535	4	9	ABCG2
SNP_A-1686878	10	8	FAS	SNP_A-1751745	4	8	ABCG2
SNP_A-1754053	10	7	PIK3AP1	SNP_A-1705909	4	8	MAPK10
SNP_A-1720407	7	6	EGFR	SNP_A-1741853	1	6	C1orf64
SNP_A-1724476	10	6	C10orf46	SNP_A-1706913	1	5	PRDM2
SNP_A-1730020	10	5	CCDC7	SNP_A-1673860	16	5	CDH13
SNP_A-1731857	10	5	BAG3	SNP_A-1697048	16	3	CDH13
SNP_A-1679064	10	4	HABP2	SNP_A-1749105	12	3	EPS8
SNP_A-1747199	7	3	EPHB6	SNP_A-1728851	10	3	MGMT
SNP_A-1674301	20	3	RBL1	SNP_A-1716085	1	3	STMN1
SNP_A-1721335	7	3	CAV2	SNP_A-1652906	4	2	IGFBP7
SNP_A-1683894	7	3	GHRHR	SNP_A-1658232	1	2	STMN1
SNP_A-1661029	7	2	NOS3	SNP_A-1721335	7	2	CAV2
SNP_A-1694743	4	2	PI4K2B	SNP_A-1651620	11	2	FGF19
SNP_A-1732612	17	2	NF1	SNP_A-1661013	16	2	CYLD
SNP_A-1695427	17	2	KSR1	SNP_A-1739981	12	2	KRAS
SNP_A-1741009	10	2	CCDC7	SNP_A-1655097	4	2	KDR
SNP_A-1720403	7	2	BRAF	SNP_A-1750171	1	2	FRAP1
SNP_A-1745332	4	2	INPP4B	SNP_A-1723196	3	2	BCL6
SNP_A-1747257	10	2	IL2RA	SNP_A-1687110	1	2	C1orf64
SNP_A-1647840	10	2	RET	SNP_A-1710047	20	2	JAG1
SNP_A-1663346	7	2	CDK6				
SNP_A-1728851	10	2	MGMT				

We annotated tag-loci that were significantly associated with more than one over- or under-expressed pathways in GBMs with their corresponding genes (GRAIL P < 0.05).

**Figure 4:**
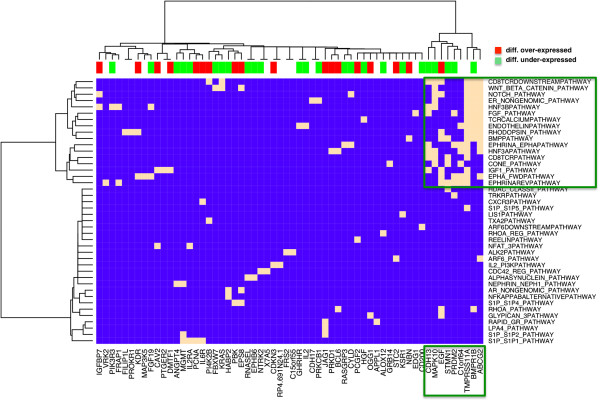
**Driver genes of under-expressed pathways.** We mapped driver genes to their corresponding, associated under-expressed pathways. Specifically, we observed a cluster of genes that was significantly associated to signaling pathways, revolving around ephrin-A/EphA related pathways. In the small cluster, we identified genes such as CDH13, EGF and MAPK10 that play important roles in neurological functions.

As for driver genes that were located nearby tag-loci associated to under-expressed pathways, we show such links between associated genes and their corresponding under-expressed pathways in a heatmap in Figure 4. Ward clustering such a matrix, we observed a small cluster of genes that largely associated with membrane based pathways revolving around ephrin-A/EphA related pathways previously linked to GBMs [33]. In the cluster of genes that were largely differentially expressed (FDR < 0.05, Student’s t-test) we found prominent cancer-related genes such as EGF. Furthermore, we found CDH13, a calcium-dependent cell–cell adhesion gene that is associated with working memory performance in attention deficit disorders and a regulator of neural cell growth [34]. Also, we observed a member of the MAP kinase family, MAPK10, that plays regulatory roles in signaling pathways during neuronal apoptosis through its phosphorylation and nuclear localization [35]. PRDM2 is a tumor suppressor gene and a member of a nuclear histone/protein methyltransferase superfamily. Although the function of this protein has not been fully characterized, it may play a role in transcriptional regulation during neuronal differentiation and pathogenesis of retinoblastoma [36]. Finally, we observed ABCG2, a membrane-associated protein that is included in the superfamily of ATP-binding cassette (ABC) transporters. Specifically, this transporter has also been shown to play protective roles in blocking absorption at the blood–brain barrier [37].

The presence of many driver genes that appear in RTK signaling prompted us to determine pathways that were enriched with such driver genes. Utilizing Fisher’s exact test, we found 14 pathways that were enriched with driver genes associated to over-expressed pathways (P < 0.05). Analogously, we obtained 12 pathways enriched with driver genes that were associated to under-expressed pathways (Table 2). Generally, such enriched pathways mainly revolved around ERBB1 signaling while PIK3 subunits and KRAS mostly drove their enrichment. Furthermore, Table 2 shows that EGFR appeared frequently among enriched pathways of genes that were associated to over-expressed pathways. In turn, EGF played this role when we focused on under-expressed pathways.

**Table 2:** Pathways enriched with driver genes that are associated to over- and under-expressed pathways in GBMs

**Over-expressed pathways**		
**Enriched pathways**	**P**	**Driver genes**
ERBB1_RECEPTOR_PROXIMAL_PATHWAY	0.001	PIK3CA KRAS EGFR GAB1
VEGFR1_PATHWAY	0.002	PGF PIK3CA NOS3 GAB1
PDGFRBPATHWAY	0.003	PTEN SHB GAB1 PTPRJ PIK3CA
TCPTP_PATHWAY	0.004	HGF PIK3CA EGFR GAB1
ERBB1_DOWNSTREAM_PATHWAY	0.005	PIK3CA EGFR GAB1 BRAF KSR1 KRAS
IL2_STAT5PATHWAY	0.019	IL2RA CDK6 PIK3CA
PI3KPLCTRKPATHWAY	0.020	PIK3CA GAB1 KRAS
ERBB1_INTERNALIZATION_PATHWAY	0.022	PIK3CA EGFR KRAS
TRKRPATHWAY	0.023	PIK3CA GAB1 NTRK2 KRAS
RET_PATHWAY	0.038	PIK3CA RET GAB1
FASPATHWAY	0.038	PIK3CA CASP3 FAS
VEGFR1_2_PATHWAY	0.039	PIK3CA SHB NOS3 GAB1
ER_NONGENOMIC_PATHWAY	0.042	PIK3CA NOS3 KRAS
TCRRASPATHWAY	0.045	BRAF KRAS
**Under-expressed pathways**		
**Enriched pathways**	**P**	**Driver genes**
ERBB2ERBB3PATHWAY	0.005	PIK3R3 MAPK10 FRAP1 KRAS
TCPTP_PATHWAY	0.006	HGF PIK3R3 EGF KDR
ET_EGFRPATHWAY	0.008	FRAP1 EGF
ERBB1_DOWNSTREAM_PATHWAY	0.009	FRAP1 PIK3R3 EPS8 KSR1 EGF KRAS
IL2_1PATHWAY	0.017	IL2RA IL2 PRKCB1 KRAS
ERBB1_RECEPTOR_PROXIMAL_PATHWAY	0.019	PIK3R3 EGF KRAS
ERBB1_INTERNALIZATION_PATHWAY	0.027	PIK3R3 EGF KRAS
TCRRASPATHWAY	0.027	PRKCB1 KRAS
CD8TCRDOWNSTREAMPATHWAY	0.033	IL2RA IL2 PRKCB1 KRAS
CXCR3PATHWAY	0.038	PIK3R3 FRAP1 KRAS
IL2_PI3KPATHWAY	0.041	IL2RA IL2 FRAP1
TELOMERASEPATHWAY	0.043	IL2 EGF FRAP1 NBN

Significantly pooling driver genes that were associated to over-expressed pathways, we found 14 pathways applying Fisher’s exact test (P < 0.05). Analogously, we observed 12 pathways enriched with driver genes that were associated to under-expressed pathways. We annotated all pathways with their corresponding driver genes.

## Discussion and conclusions

We applied a stepwise methodology to uncover genomic alterations that are informative of observed patterns of pathway activity changes in glioblastoma multiforme, providing a high-level picture of the cell’s molecular phenotype. Usually, association studies suffer from a large number of tests, contributing to a massive multiple testing problem. In our case, we mitigated this issue by using a limited number of tag-loci. Furthermore, a low number of tested pathways contributed to lower statistical complexity as well, limiting the number of applied tests.

While others have investigated the influence of copy number alterations on gene expression in GBMs before, such studies focused on single genes [38] to identify regulatory networks. Furthermore, other authors used network-based approaches involving genes that were placed in areas of copy number alterations to identify candidate oncogenic, modular processes and driver genes [12]. Here, we investigated patterns that emerge from large-scale genomic eQTL-like associations to whole groups of genes. In particular, we represented each pathway by its corresponding leading edge genes, defined as subset of genes that govern the over/under expression of a pathway, comparing disease to control cases. Applying a non-linear eQTL approach we observed that genomic signature alterations of GBMs largely translated into elevated normalized importance scores of corresponding tag-loci and high frequencies of associated pathways. As for over-expressed pathways, significantly associated tag-loci were largely limited to chromosomes 7 and 10, an expected result since alterations on chromosomes 7 and 10 belong to signature modifications in GBMs. Surprisingly, we observed the emergence of chromosome 4 as the major contributor of associations to under-expressed pathways while associations to tag-loci on chromosomes 7 and 10 were largely absent. Such an observation was rather unexpected as chromosome 4 lacks frequent copy number alterations, while its involvement has been shown only in a subset of GBMs [39]. Furthermore, we also found that composite effects between chromosomes that are associated to under-expressed pathways also involved a variety of other genomic locations. In turn, such observations remained limited to tag-loci on chromosomes 7 and 10 that were associated to over-expressed pathways.

Since genomic regions that were found to be frequently associated to pathways referred to known alterations, we performed an analysis of the relatedness of genes based on disease regions in gliomas, allowing us to identify potential driver genes. Qualitatively, we observed that some genes were already identified as driver genes in GBMs, indicating the relevance of the determined associations. Furthermore, we identified a small set of driver genes that were associated to under-expressed pathways. While such a set included EGF as a prominent driver gene we also found a variety of genes that have important neuronal functions. While their involvement in such a cluster suggests a composite effect with EGF, their prevalence in associations to under-expressed pathways may indicate a previously unknown role in GBMs as well.

While we observed that many observed driver genes were included in prominent signaling and regulation pathways we determined pathways that were enriched with such genes. Since we considered associations to signaling pathways such driver pathways may represent a core that governs the change of the signaling apparatus in GBM. In particular, we found 14 pathways that were enriched with genes associated to over-expressed pathways. Specifically, PIK3 subunits, KRAS and EGFR were frequently involved in such pathways. In turn, we obtained 12 pathways enriched with genes that were associated to under-expressed pathways. While PIK3 subunits and KRAS were involved in these pathways too, we frequently found EGF instead of EGFR, an interesting observation given that the interaction of EGF and EGFR triggers many important signaling and regulation processes in human cancers.

These observed patterns of common and diverging genomic regions may indicate that a rational design of drug combinations for the treatment of brain tumors follows similar patterns of common and diverging alterations, generally pointing to avenues for the design of glioma subtype specific drug cocktails. In particular, our results suggest that therapy approaches may target different pathways simultaneously. Indeed, combination therapy with EGFR inhibitors [40] and drugs targeting the PI3K/AKT/PTEN pathway [41] were considered for the design of GBM specific drug cocktails.

Currently, we only accounted for genomic alterations, omitting other potential molecular causes for the emergence of GBMs. Further analysis of associated pathways will have to include other sources of molecular genome-wide data. For example, methylation data may indicate other avenues that contribute to the expression regulation of pathways. Therefore, the integration of such data as variables may allow us to identify composite effects between methylation characteristics and genomic alterations that can influence the expression change of pathways and point to novel, previously unknown regulation mechanisms.

## Methods

### mRNA treatment

We investigated 158 glioblastoma multiforme patient and 21 non-tumor control samples from epilepsy patients from the Rembrandt database (https://caintegrator.nci.nih.gov/caintegrator/) that were collected from the NCI-sponsored Glioma Molecular Diagnostic Initiative (GMDI) and from Henry-Ford hospital (HF) [13,25]. Using HG-U133 Plus 2.0 arrays, normalization was performed at the PM and MM probe level with dChip [25,42]. Using the average difference model to compute expression values, model-based expression levels were calculated with normalized probe level data, and negative average differences (MM > PM) were set to 0 after log-transforming expression values [25]. Accounting for weak signal intensities, all probe sets with more than 10% of zero log-transformed expression values were removed. To represent a gene, we chose the corresponding probeset with the highest mean intensity in each tumor subtype.

### Determination of copy number alterations

Matching patient genomic data were collected from the Rembrandt database (https://caintegrator.nci.nih.gov/caintegrator/) where all samples were hybridized on Genechip Human Mapping 100 K arrays. Copy numbers were calculated using Affymetrix Copy Number Analysis Tool (CNAT 4). After probe-level normalization and summarization calculated log_2_-tranformed ratios were used to estimate raw copy numbers. Using a Gaussian approach raw SNP profiles were smoothed (> 500 kb window by default) [13,43,44].

### Detection of Tag-loci

We represented each patient sample as a set of loci, *L* = {*l*_1_, *l*_2_, …, *l*_*m*_}, where each locus *l*_*i*_ was characterized by the corresponding copy number *cn*_*i,j*_ in each case *j*, *CN*_*i*_ = {*cn*_*i*,1_, *cn*_*i*,2_, …, *cn*_*i*,*n*_}. Since copy numbers of nearby loci tend to be highly correlated we significantly reduced the number of loci by a local clustering. For a potential tag-locus *tl*_*k*_, we greedily accumulated all consecutive loci, ensuring that the Pearson’s correlation coefficient of *CN*_*k*_ and *CN*_*i*_ at any locus *l*_*i*_ in the region was *>*0.95*.* Since adjacent regions overlap a gene may belong to more than one region [24].

### Random forests

Random Forests is an ensemble learning method [45] where regression and classification trees are constructed using *N* different bootstrap samples of the data (‘bagging’). In addition, random forests change how regression trees are constructed by splitting each node, using the best among a subset of *M* randomly chosen predictors (‘boosting’). New data is predicted by aggregating the predictions of *N* trees. As for our regressions, we used $\sqrt{n}$ of all *n* tag-loci and randomly picked $\sqrt{x}$ of all *x* samples for the construction of each of *N =* 1,000 trees. As output, random forests provide an importance score that reflects the increase of the prediction error when the given locus is omitted in the regression process.

## Competing interests

The authors declare no competing interests.

## Authors’ contributions

SW and AV designed the analysis. SW generated data. SW, AV, SB and POB analyzed data and wrote the paper. All authors read and approved the final manuscript.

## Pre-publication history

The pre-publication history for this paper can be accessed here:

http://www.biomedcentral.com/1755-8794/6/11/prepub

## Supplementary Material

Additional file 1: Table S1 List of all significantly associated pairs of over- and under expressed pathways and tag-loci (FDR < 0.05). Click here for file

Additional file 2: Table S2 Common molecular alterations (mutations, amplifications and/or deletions) in gliomas. Molecular alterations are indicated by the corresponding literature references. Click here for file

Additional file 3: Table S3 List of all associated tag-loci in GBMs, their corresponding number of over- and under-expressed, associated pathways and genes placed nearby tag-loci that are significantly similar to common molecular alterations in gliomas (P < 0.05). Click here for file
