# Supplementary material for: Genome-wide associations of signaling pathways in glioblastoma multiforme
Source: BMC Med Genomics. 2013 Mar 28;6:11. doi: 10.1186/1755-8794-6-11 (PMC3616958; doi:10.1186/1755-8794-6-11)
Supplement: Additional file 2: Table S2 — Common molecular alterations (mutations, amplifications and/or deletions) in gliomas. Molecular alterations are indicated by the corresponding literature references. [file 1755-8794-6-11-S2.docx]

| **Gene** | **GBM** | **Oligos** |
| --- | --- | --- |
| EGFR | ([1](#_ENREF_1), [2](#_ENREF_2)) | ([3](#_ENREF_3)) |
| CDK4 | ([1](#_ENREF_1), [2](#_ENREF_2)) | ([4](#_ENREF_4)) |
| PDGFRA | ([1](#_ENREF_1)) |  |
| MDM2 | ([1](#_ENREF_1), [2](#_ENREF_2)) | ([4](#_ENREF_4)) |
| MDM4 | ([1](#_ENREF_1), [2](#_ENREF_2)) |  |
| MET | ([1](#_ENREF_1)) |  |
| CDK6 | ([1](#_ENREF_1), [2](#_ENREF_2)) |  |
| MYCN | ([1](#_ENREF_1)) |  |
| CCND2 | ([1](#_ENREF_1), [2](#_ENREF_2)) |  |
| PIK3CA | ([1](#_ENREF_1), [2](#_ENREF_2)) |  |
| AKT3 | ([1](#_ENREF_1)) |  |
| CDKN2A | ([1](#_ENREF_1)) | ([3](#_ENREF_3), [4](#_ENREF_4)) |
| CDKN2B | ([1](#_ENREF_1)) |  |
| PTEN | ([1](#_ENREF_1), [2](#_ENREF_2)) | ([3](#_ENREF_3)) |
| CDKN2C | ([1](#_ENREF_1)) |  |
| RB1 | ([1](#_ENREF_1), [2](#_ENREF_2)) |  |
| PARK2 | ([1](#_ENREF_1)) |  |
| NF1 | ([1](#_ENREF_1), [2](#_ENREF_2)) |  |
| TP53 | ([1](#_ENREF_1), [2](#_ENREF_2)) | ([4](#_ENREF_4), [5](#_ENREF_5)) |
| ERBB2 | ([1](#_ENREF_1)) |  |
| PIK3R1 | ([1](#_ENREF_1), [2](#_ENREF_2)) |  |
| IDH1 | ([2](#_ENREF_2)) | ([2](#_ENREF_2)) |

**Table S2:** Common molecular alterations (mutations, amplifications and/or deletions) in gliomas. Molecular alterations are indicated by the corresponding literature references.

1. Comprehensive genomic characterization defines human glioblastoma genes and core pathways. Nature. 2008;455:1061-8.

2. Ohgaki H, Kleihues P. Genetic alterations and signaling pathways in the evolution of gliomas. Cancer Sci. 2009;100:2235-41.

3. Reifenberger G, Louis DN. Oligodendroglioma: toward molecular definitions in diagnostic neuro-oncology. J Neuropathol Exp Neurol. 2003;62:111-26.

4. Watanabe T, Yokoo H, Yokoo M, Yonekawa Y, Kleihues P, Ohgaki H. Concurrent inactivation of RB1 and TP53 pathways in anaplastic oligodendrogliomas. J Neuropathol Exp Neurol. 2001;60:1181-9.

5. Okamoto Y, Di Patre PL, Burkhard C, Horstmann S, Jourde B, Fahey M, et al. Population-based study on incidence, survival rates, and genetic alterations of low-grade diffuse astrocytomas and oligodendrogliomas. Acta Neuropathol. 2004;108:49-56.
